# Cardiohistological Findings in Refeeding Syndrome

**DOI:** 10.7759/cureus.67430

**Published:** 2024-08-21

**Authors:** Toru Miyoshi, Haruhiko Higashi, Kisaki Amemiya, Yoshihiko Ikeda, Osamu Yamaguchi

**Affiliations:** 1 Department of Cardiology, Pulmonology, Hypertension and Nephrology, Ehime University Graduate School of Medicine, Toon, JPN; 2 Department of Pathology, National Cerebral and Cardiovascular Center, Suita, JPN

**Keywords:** adipose triglyceride lipase, anorexia nervosa, histological findings, takotsubo syndrome, refeeding syndrome

## Abstract

Severe eating disorders may develop refeeding syndrome, which sometimes resembles severe cardiac dysfunction. A woman in her thirties was admitted to our hospital because of cardiogenic shock. Transthoracic echocardiography showed reduced left ventricular systolic function. In her medical history, she had been diagnosed with refeeding syndrome. A ventricular endocardial biopsy was performed to exclude other cardiac diseases. A histological examination showed conspicuously atrophied cardiomyocytes with nuclear swelling and irregularities; the myocardial sequence was disturbed with fibrosis. Immunostaining revealed that lipid droplet markers, adipose triglyceride lipase, and perilipin 2 were poorly observed in the cardiomyocytes, while expression of both proteins was attenuated in fibroblasts within the myocardial layer. The abnormal metabolism of fatty acids was the presumed cause of cardiac dysfunction.

## Introduction

Refeeding syndrome is a potentially fatal condition in which massive electrolyte shifts occur in the body resulting from rapid feeding after a prolong period of low caloric intake [1]. Patients with anorexia nervosa (AN) often have inadequate caloric intake, which can lead to refeeding syndrome. Hypophosphatemia, hypomagnesaemia, and hypokalaemia often occur in refeeding syndrome after the initiation of general IV therapy, with potentially fatal consequences such as heart, respiratory, and multiple organ failure. Hypophosphetemia is responsible for the development of refeeding syndrome. Hypophosphataemia in the blood reduces 2,3-diphosphoglycerate in erythrocytes, which is associated with the oxygen affinity of hemoglobin [2]. The oxygen supply to peripheral tissues is reduced because the oxygen affinity of hemoglobin is decreased. In addition, peripheral tissues are also depleted of phosphorus, resulting in decreased adenosine triphosphate (ATP). It causes organ damage from energy deprivation [3,4].

We report the case of a patient with AN who developed refeeding syndrome complicated by takotsubo syndrome, in which a myocardial biopsy demonstrated impaired fatty acid metabolism.

## Case presentation

A woman in her thirties was admitted to the prior hospital because of disturbed consciousness and a Glasgow Coma Scale score of 3 on arrival. She was hypoglycemic and promptly administered a 50% dextrose solution intravenously three days ago. She had been suffering from AN for one year and had a BMI of 13 kg/m^2^ upon arrival. She developed hypophosphatemia after a proper meal and was diagnosed with refeeding syndrome secondary to AN. Subsequently, the patient developed hypotension, dyspnea, and was diagnosed with acute heart failure. Her blood pressure remained critically low at 65/33 mmHg despite the administration of dopamine 10 μg/kg/min and noradrenaline 0.2 µg/kg/min. She was subsequently diagnosed with cardiogenic shock. She was transferred to our hospital due to prolonged cardiogenic shock.

Her blood test results were as follows: leukocytes, 7,400/μL; aspartate aminotransferase (AST), 285 U/L; alanine aminotransferase (ALT), 662 U/L; potassium, 3.4 mmol/L; phosphate, 1.3 mg/dL (reference range, 2.2-5.3); brain natriuretic peptide (BNP), 1,022 pg/mL; troponin T, 58 pg/mL(reference range, ≤ 50); and vitamin B1, 22 pg/mL (reference range, 24-66)（Table 1).

**Table 1:** Blood tests at the time of transfer The patient's blood tests showed hypophosphatemia, low vitamin B1, and elevated AST, ALT, and BNP levels. AST: Aspartate aminotransferase; ALT: Alanine aminotransferase; BUN: Blood urea nitrogen; BNP: Brain natriuretic peptide

Parameters	Result	Reference Range
Leukocytes	7,400/μL	3,500-9,100/μL
Hemoglobin	10.5 g/dL	11.3-15.2 g/dL
Platelet Count	17.8 × 10^4^/μL	13.1-36.9 × 10^4^/μL
AST	285 U/L	9-37 U/L
ALT	662 U/L	3-49 U/L
BUN	20 mg/dL	7-21 mg/dL
Creatinine	0.38 mg/dL	0.5-1.2 mg/dL
Na (Sodium)	136 mmol/L	139-149 mmol/L
K (Potassium)	3.4 mmol/L	3.8-4.8 mmol/L
Phosphate	1.3 mg/dL	2.2-5.3 mg/dL
BNP	1,022 pg/mL	≤ 18.4 pg/mL
Troponin T	58 pg/mL	≤ 50 pg/mL
Vitamin B1	22 pg/mL	24-66 pg/mL

**Figure 1:**
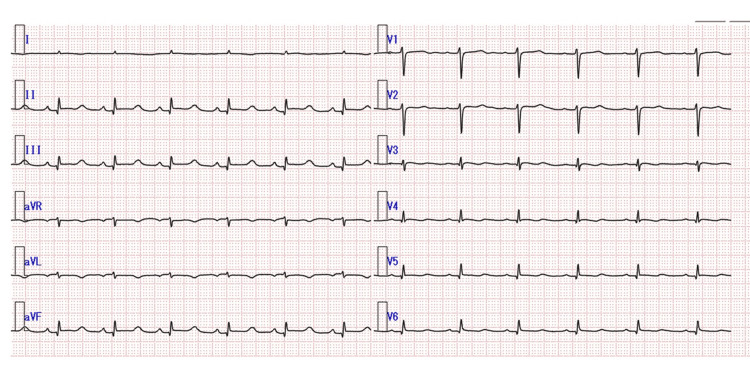
Electrocardiogram Electrocardiogram taken at admission. The patient's ECG findings showed ST-segment elevation in the II, III, aVF leads.

Electrocardiography showed sinus rhythm (75 beats per minutes) with low voltage in the precordial leads and ST elevation in leads II, III, and aVF. The QRS width was 120 ms (Figure 1).

**Figure 2:**
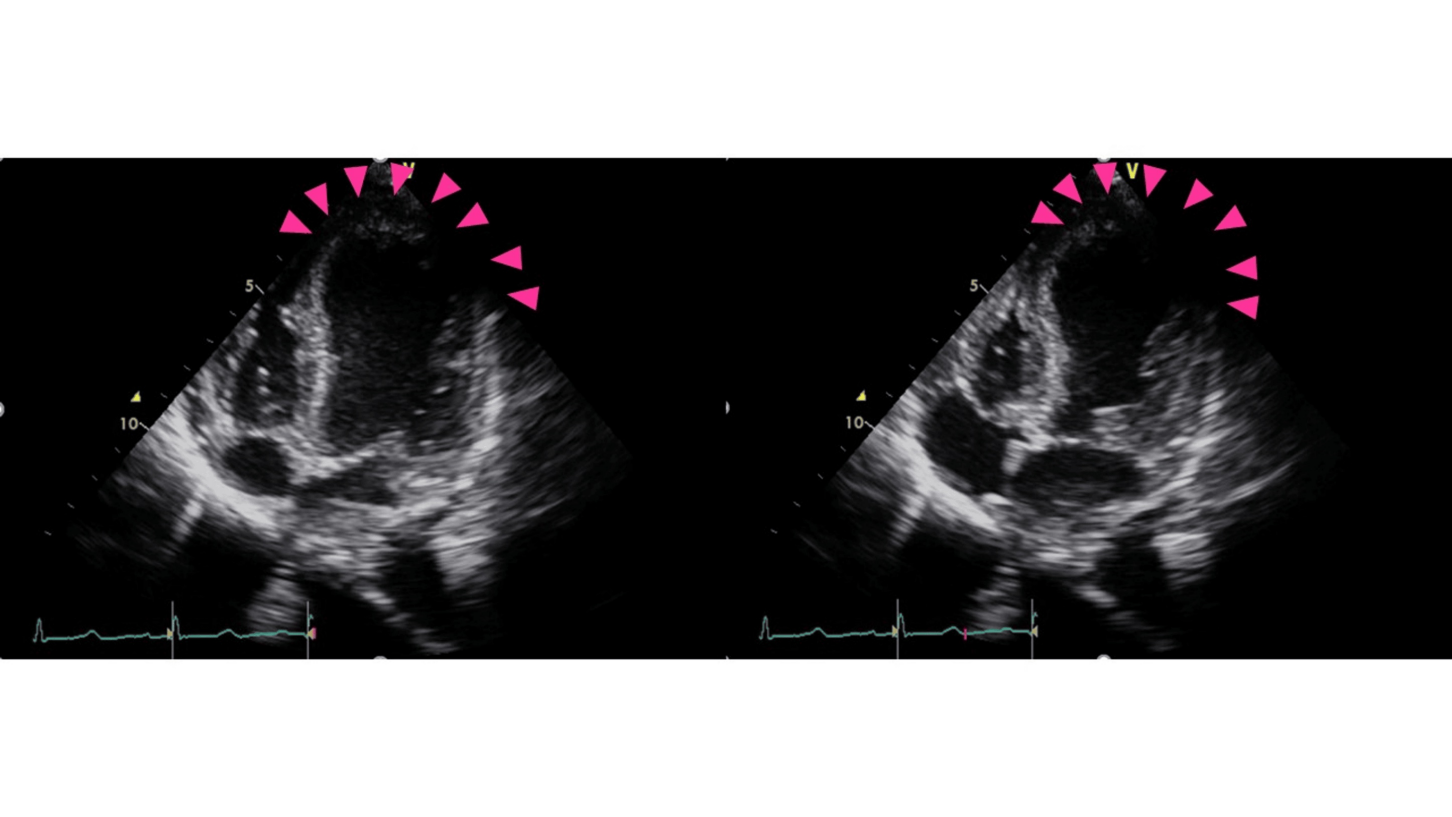
Transthoracic echocardiography at admission Left panel: Diastolic phase; Right panel: Systolic phase

Transthoracic echocardiography showed the end-diastolic diameter of the left ventricle as 42 mm, the left ventricular ejection fraction as 27%, and basal wall motion was maintained. However, akinesis was observed from the middle to the apex, suggesting takotsubo syndrome (Figures 2A and B).

Coronary CT angiography (CTCA) revealed no significant stenosis of the coronary arteries. Right heart catheterization was performed under inotropic support. The pulmonary artery wedge pressure was found to be 7 mmHg, mean pulmonary artery pressure was 11 mmHg, right atrial pressure was 4 mmHg, and cardiac index was 1.7 L/min/m^2^. An endomyocardial biopsy of the right ventricular septum was performed immediately after admission to rule out myocarditis.

**Figure 3:**
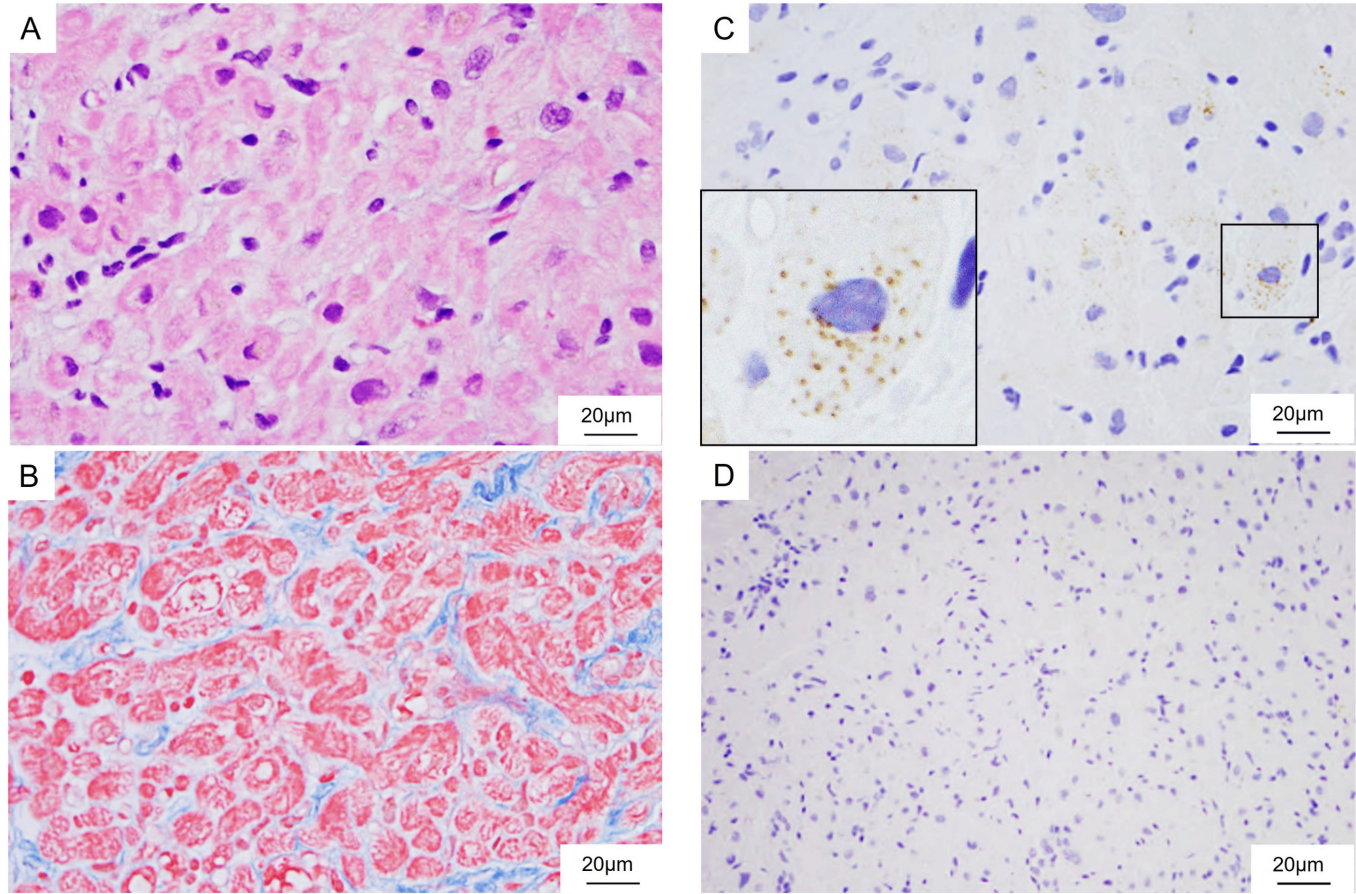
Endomyocardial biopsy histology and immunohistochemistry (A) Hematoxylin and eosin staining; (B) Masson’s trichrome staining; (C) Immunostaining for adipose triglyceride lipase; (D) Immunostaining for perilipin 2

A histological examination showed atrophic cardiomyocytes with moderate myocardial interstitial fibrosis. No inflammatory infiltrates were observed. Therefore, we concluded that myocarditis was unlikely. On the other hand, adipose triglyceride lipase (ATGL), the lipolytic rate-limiting enzyme of adipocytes, was partially expressed in the myocardium, which indicated no ATGL deficiency. Perilipin 2, a well-known protein that coats lipid droplets in multiple nonadipose tissues, was negative, which indicated no deposition of lipid droplets in the cardiomyocytes (Figure 3).

**Figure 4:**
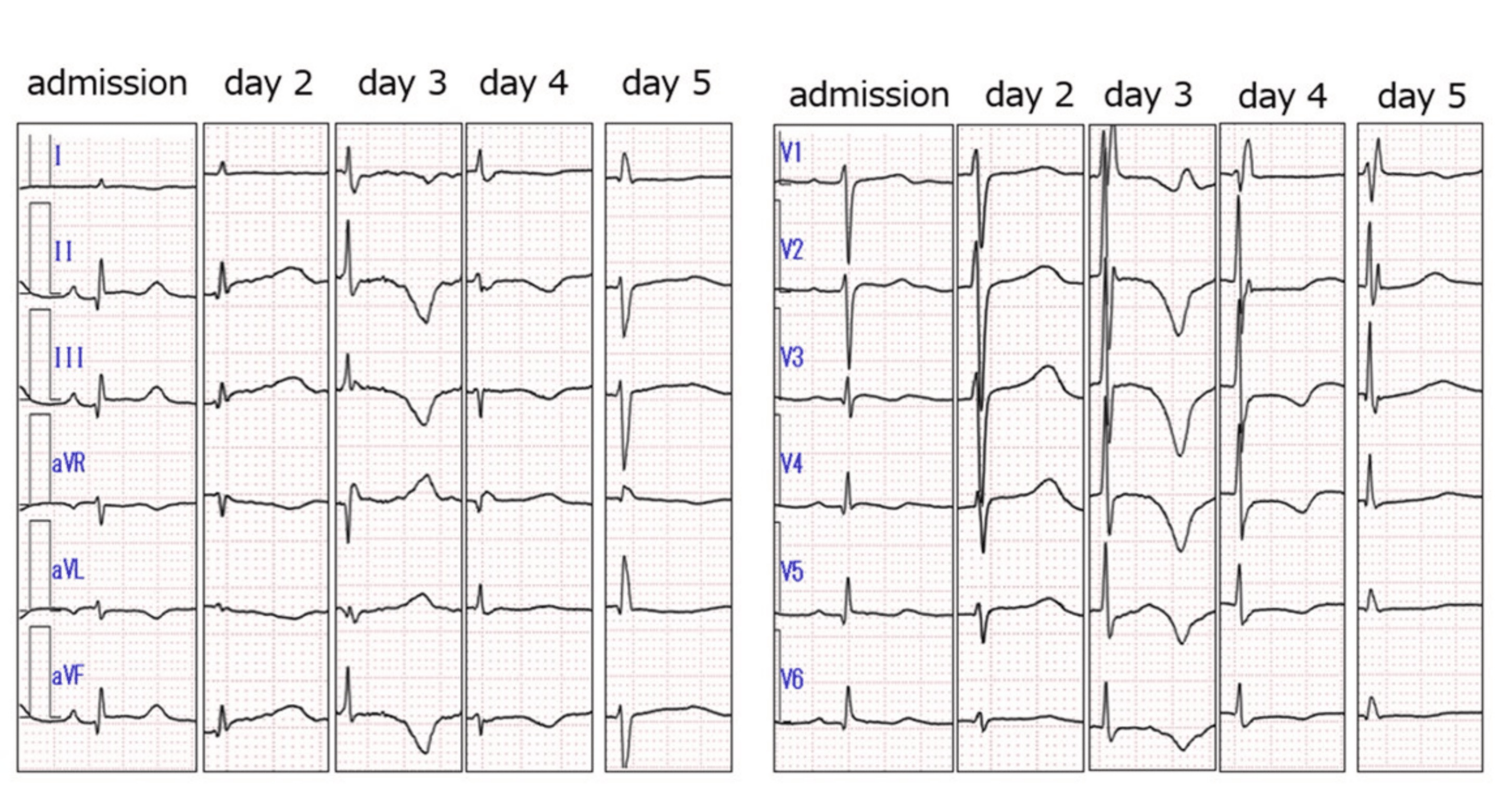
Electrocardiogram follow-up In electrocardiogram follow-up, her ECG  showed a giant negative T wave on the third day of admission that gradually improved.

Electrocardiogram on admission showed a giant negative T wave on the third day of admission that gradually improved (Figure 4).

Supplementation with vitamin B1 and trace elements was initiated, and a total caloric count of 700 kcal/day was maintained along with monitoring of blood glucose level. The left ventricular wall motion gradually improved. Her left ventricular wall movement improved, and dobutamine infusion was discontinued. The patient’s daily caloric intake was increased by 200 kcal/day with one week of monitoring, and she was discharged once the caloric intake reached 1,600 kcal/day.

Just before discharge, the patient’s heart failure improved to New York Heart Association class I, and echocardiography showed an improved left ventricular end-diastolic diameter of 40 mm and left ventricular ejection fraction of 72%. A psychiatric consultation after discharge led to improvement in the patient’s eating disorder and an increase in her BMI to 16 kg/m^2^.

## Discussion

To the best of our knowledge, this case was the first case of a histological demonstration of an endomyocardial biopsy performed in a patient with refeeding syndrome. Refeeding syndrome often associated with severe AN and may present with hypoglycemia and hypophosphatemia, as well as cardiogenic shock after feeding [5]. Friedli et al. reported that mortality rate was as high as 29.8% [6].

The present case had severe AN and was diagnosed with refeeding syndrome after the development of hypophosphatemia and hypoglycemia postprandially [7]. Histological analysis of the myocardial biopsy specimen demonstrated cardiomyocyte atrophy and findings suggestive of impaired fatty acid metabolism. Immunostaining showed positive reactivity for perilipin 2, a lipid droplet marker that is otherwise scarce in cardiomyocytes [8]. Positive reactivity for ATGL, a rate-limiting enzyme for intracellular neutral lipolysis, was also scarce in a small population of cardiomyocytes [9]. The impairment of lipid storage and lipolysis in cardiomyocytes was histologically demonstrated in this case. Although it was enhanced and the in vivo reaction toward lipolysis metabolism was active, the abnormal metabolism of fatty acids, an energy source for cardiomyocytes, may have caused the cardiac dysfunction. Myocardial tissue uses glucose and fatty acids as energy sources. Glucose stores are depleted and fatty acids are used as energy sources in cases of severe nutritional disorders, such as neuropathic anorexia.

The pathogenesis of takotsubo syndrome is still controversial, but it has been reported that the possibility that catecholamines may be involved. The rapid increase in glucose metabolism caused by nutritional supplementation after prolonged starvation causes hypersecretion of catecholamines and supply of an abnormal energy such as fatty acids, resulting in takotsubo syndrome. This was confirmed by myocardial histopathology with impaired function caused by fatty acid depletion secondary to refeeding syndrome.

Refeeding syndrome with cardiac dysfunction is not uncommon in intensive care situation. Systolic dysfunction due to Refeeding syndrome may be complicated by takotsubo syndrome due to elevated catecholamines and should be treated with caution. Patients with severe eating disorders may develop refeeding syndrome with takotsubo syndrome due to the depletion of long-chain fatty acids in the myocardium.

## Conclusions

We experienced a case of takotsubo syndrome after a short period of re-nutrition in response to prolonged starvation. To the best of our knowledge, this is the first report of a refeeding syndrome, which consists of myocardial pathology. Patients with severe eating disorders may develop a refeeding syndrome with takotsubo syndrome due to depletion of long-chain fatty acids in the myocardium.

## References

[1] Collins S (1995). The limit of human adaptation to starvation. Nat Med.

[2] MacDonald R (1977). Red cell 2,3-diphosphoglycerate and oxygen affinity. Anaesthesia.

[3] O'Connor G, Nicholls D (2013). Refeeding hypophosphatemia in adolescents with anorexia nervosa: a systematic review. Nutr Clin Pract.

[4] Ariyoshi N, Nogi M, Ando A, Watanabe H, Umekawa S (2016). Hypophosphatemia-induced cardiomyopathy. Am J Med Sci.

[5] Shimizu K, Ogura H, Wasa M, Hirose T, Shimazu T, Nagasaka H, Hirano K (2014). Refractory hypoglycemia and subsequent cardiogenic shock in starvation and refeeding: report of three cases. Nutrition.

[6] Friedli N, Baumann J, Hummel R (2020). Refeeding syndrome is associated with increased mortality in malnourished medical inpatients: secondary analysis of a randomized trial. Medicine (Baltimore).

[7] Skowrońska A, Sójta K, Strzelecki D (2019). Refeeding syndrome as treatment complication of anorexia nervosa. Psychiatr Pol.

[8] Sztalryd C, Brasaemle DL (2017). The perilipin family of lipid droplet proteins: gatekeepers of intracellular lipolysis. Biochim Biophys Acta Mol Cell Biol Lipids.

[9] Schreiber R, Xie H, Schweiger M (2019). Of mice and men: the physiological role of adipose triglyceride lipase (ATGL). Biochim Biophys Acta Mol Cell Biol Lipids.

